# Naturally Formed Chitinous Skeleton Isolated from the Marine Demosponge *Aplysina fistularis* as a 3D Scaffold for Tissue Engineering

**DOI:** 10.3390/ma14112992

**Published:** 2021-06-01

**Authors:** Tomasz Machałowski, Agnieszka Rusak, Benita Wiatrak, Katarzyna Haczkiewicz-Leśniak, Aneta Popiel, Jakub Jaroszewicz, Andrzej Żak, Marzenna Podhorska-Okołów, Teofil Jesionowski

**Affiliations:** 1 Institute of Chemical Technology and Engineering, Faculty of Chemical Technology, Poznan University of Technology, 60-965 Poznan, Poland; tomasz.g.machalowski@doctorate.put.poznan.pl; 2 Department of Histology and Embryology, Faculty of Medicine, Wroclaw Medical University, Chalubinskiego 6a, 50-368 Wroclaw, Poland; popielaneta1@gmail.com; 3 Department of Pharmacology, Faculty of Medicine, Wroclaw Medical University, J. Mikulicza-Radeckiego 2, 50-345 Wroclaw, Poland; benita.wiatrak@umed.wroc.pl; 4 Department of Basic Medical Sciences, Faculty of Pharmacy, Wroclaw Medical University, Borowska 211, 50-556 Wroclaw, Poland; 5 Department of Ultrastructural Research, Faculty of Medicine, Wroclaw Medical University, Chalubinskiego 6a, 50-368 Wroclaw, Poland; katarzyna.haczkiewicz@umed.wroc.pl (K.H.-L.); marzenna.podhorska-okolow@umed.wroc.pl (M.P.-O.); 6 Faculty of Materials Science and Engineering, Warsaw University of Technology, 02-507 Warsaw, Poland; jakub.jaroszewicz@pw.edu.pl; 7 Electron Microscopy Laboratory, Faculty of Mechanical Engineering, Wroclaw University of Science and Technology, Wybrzeze Wyspianskiego 27, 50-370 Wroclaw, Poland; andrzej.zak@pwr.edu.pl

**Keywords:** chitin, scaffolds, fibroblasts, keratinocytes, neurons

## Abstract

Tissue engineering (TE) is a field of regenerative medicine that has been experiencing a special boom in recent years. Among various materials used as components of 3D scaffolds, naturally formed chitinous materials seem to be especially attractive because of their abundance, non-toxic and eco-friendly character. In this study, chitinous skeleton isolated from the marine sponge *Aplysina fistularis* (phylum: Porifera) was used for the first time as a support for the cultivation of murine fibroblasts (Balb/3T3), human dermal fibroblasts (NHDF), human keratinocyte (HaCaT), and human neuronal (SH-SY5Y) cells. Characterization techniques such as ATR FTIR, TGA, and μCT, clearly indicate that an interconnected macro-porous, thermostable, pure α-chitin scaffold was obtained after alkali–acid treatment of air-dried marine sponge. The biocompatibility of the naturally formed chitin scaffolds was confirmed by cell attachment and proliferation determined by various microscopic methods (e.g., SEM, TEM, digital microscopy) and specific staining. Our observations show that fibroblasts and keratinocytes form clusters on scaffolds that resemble a skin structure, including the occurrence of desmosomes in keratinocyte cells. The results obtained here suggest that the chitinous scaffold from the marine sponge *A. fistularis* is a promising biomaterial for future research about tissues regeneration.

## 1. Introduction

Natural polymers (biopolymers) isolated from renewable resources, such as plants, animals, or microorganisms, are attractive propositions of scaffolds substrates [1]. Because of their strong similarity to the extracellular matrix (ECM), the use of such materials may eliminate chronic inflammation, toxicity, or immune system response, commonly recorded through synthetic polymers [2]. Recently, frequently used are natural bioscaffolds based on proteins (e.g., collagen, silk, or gelatin), polysaccharides (e.g., alginate, hyaluronic acid, or chitosan), or their combinations [2]. As an example, Baldino et al. described an innovative method of producing alginate-chitosan aerogels for biomedical application through supercritical drying [3]. As observed, chitin has also been intensively developed with respect to biomedical engineering application [4,5,6,7,8]. Thanks to its abundance and extraordinary features, such as biocompatibility and biodegradability, this biopolymer is used in the fields of tissue engineering (TE) [9,10]. It should be stated that chitin also present cohesive interaction with blood components, e.g., erythrocytes, IgG, fibrinogen (beneficial for preventing side-effects), and nontoxicity [11]. Nevertheless, for creating an effective scaffold, other parameters must also be provided, such as advantageous 3D geometrical conformation, porosity, wettability, surface roughness, and suitable nano- and micromechanical properties [12]. Moreover, because of its nanoscale fibrous morphology, chitin has been found to be a particularly valuable biological material that accelerates wound healing processes, reduces scarring, and offers antibacterial properties [8]. However, crucial variances exist in the properties, microstructure, and processability of chitins from commercial sources, such as crustaceans or fungi, affecting their application in wound treatment. On the other hand, the use of chitin causes many problems during the creation of 3D scaffolds. Firstly, chitin is insoluble in conventional solvents because of its abundant inter- and intra-sheet hydrogen bonds and crystalline areas [13]. In the literature, we can find only a few solvents for the dissolution of chitin, e.g., hexafluoroisopropyl alcohol (HFIP), a dimethylacetamide (DMA)–LiCl mixture, CaCl_2_–MeOH mixture, phosphoric acid, or ionic liquids [14]. Secondly, commercial chitin (from crustaceans and fungi) is obtained as granules, flakes, sheets, or powder, not as the two- or three-dimensional scaffolds required for TE applications [15]. Thus, to avoid costly processing, there is an urgent need to seek naturally formed chitinous constructs. Recently, two key routes of scaffold fabrication have been developed. The first involves the production of matrices with previously designed three-dimensional architecture, e.g., by 3D printing [16,17,18,19], macro-porous hydrogels synthesis [20,21,22], or electrospinning [23,24]. The second uses renewable decellularized natural sources of prefabricated scaffolds in their original shape, e.g., autogenic or allogenic ECM [25,26,27], skeletons with proper architecture [28,29] or plant-based structure [30].

Marine sponges are a remarkable donors of renewable biological materials [28,31]. Especially Verongida (Porifera) in particular was established as a good potential source of bioactive compounds (e.g., aeroplysinin-1 or isofistularin-3) [4,32,33] and chitinous skeletons [34,35,36,37,38]. Moreover, the possibility of underwater sponge farming makes them even more attractive [4]. The first discovery of chitinous skeletons in the marine sponge was in 2007 [39]. These investigations were fascinating from an evolutionary perspective, particularly because they indicated that chitin appeared several hundred million years before those in arthropod skeletons [28]. The fibrous skeletons are made of α-chitin, which forms a biocomposite with inorganic salts (calcium- and silica-based) and bromotyrosines. In nature, such constructs are essential for a sponge’s mechanical rigidity and for chemical defense against predators [29]. The purified chitinous scaffold obtained after decellularization and demineralization processes has been recognized as a unique microenvironment for cells. As previously described, such constructs have promoted cell and metabolite migration in the cultivation of human mesenchymal stromal cells (hMSCs) [40,41,42] and cardiomyocytes [4,34]. It should be stated that to compare with other “chitin sources”, only sponges create a tube-like, interconnected, macro-porous, and structurally organized 3D chitinous matrix. Thus, after well-conducted isolation, a “ready to use” product could be obtained. Moreover, the ability to cultivate sponges under marine farming conditions makes this material especially attractive to compare with other natural and synthetic substrates. Inspired by the above findings, we attempted to use naturally formed chitinous scaffolds from *A. fistularis* marine sponge, which have the appropriate shape, size, and porosity for structural support in cell cultivation. The cultures were lines of murine fibroblasts (Balb/3T3), human fibroblasts (NHDF), human keratinocytes (HaCaT), and neural cells (SH-SY5Y). The cytotoxicity effect, cell distribution, and adhesion were assessed. When choosing a material to fill, e.g., bone damage, it is important that the material also provides an appropriate environment for the surrounding nerves. In the case of damage to peripheral nerves, bioactivity and biocompatibility with respect to their regeneration and the correct direction of axonal growth was demonstrated. The obtained results show that chitin-based scaffolds of poriferan origin are promising materials for future tissue engineering applications, particularly as artificial skin or dressing materials.

## 2. Materials and Methods

### 2.1. Preparation of Chitinous Scaffolds

**Figure 1:**
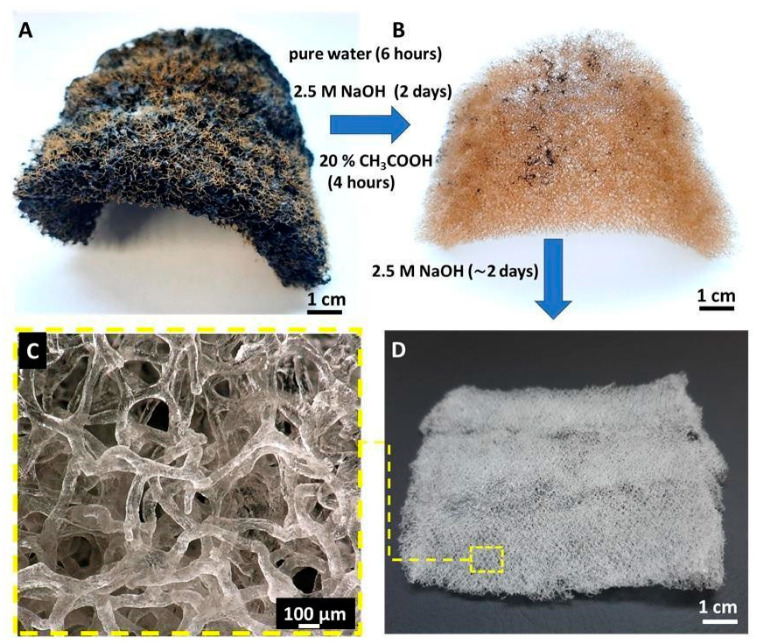
(**A**) Dried fragment of *A. fistularis* marine sponge. (**B**) Chitin-based cell-free skeleton becomes isolated after the first alkali-based treatment. (**C**,**D**) Purified 3D chitinous scaffolds which resembles micro-tubular shape and size of natural sponge skeleton.

Chitin-based scaffolds were isolated by chemical treatment of air-dried *A. fistularis* sponge skeleton, with a total isolation time of around 5 days (see Figure 1). At the first step, fragments of sponge skeleton were submerged in deionized water for 6 h to dispose of water-soluble impurities. The skeletons were then immersed in 2.5 M sodium hydroxide solution for 48 h for decellularization and deproteinization at 36 °C. The 3D scaffolds were neutralized with deionized water and treated for 4–5 h with 20% acetic acid as a demineralization agent. At the pre-final stage, 2.5 M NaOH solution was again used at the same conditions until pure chitin was isolated. Finally, colorless tubular chitin scaffolds were neutralized with distilled water and then kept in 70% ethanol at 4 °C.

### 2.2. Chitin Scaffold Sterilization and Preparation

Pure chitinous scaffolds were sterilized using a high-temperature program for the sterilization of liquids (121 °C). Sterilization was carried out in sterile PBS using a SterilClave autoclave, model 18B, from Cominox (Como, Italy). The sterilized scaffolds were then stored in a pH = 7.4 PBS solution at 4 °C. Finally, the scaffolds were shifted to a sterile 6-well cell culture plate and incubated into the culture medium with cells.

### 2.3. Characterization Techniques

#### 2.3.1. Digital Microscopy

Preparations were visualized using an advanced microscope set consisting of a Keyence VHX-7000 digital optical microscope (Osaka, Japan) and VH-Z20R swing-head zoom lenses (maximal magnification 200×).

#### 2.3.2. Attenuated Total Reflectance–Fourier Transformation Infrared Spectroscopy (ATR FTIR)

The ATR FTIR infrared spectroscopy technique (attenuated total reflectance) was used for the characterization of the isolated scaffolds and determination of their purity. The presence of characteristic moieties was detected using a VERTEX 70 spectrometer (Bruker, Karlsruhe, Germany). The wide wavenumber range of 4000–400 cm^−1^ (resolution 0.5 cm^−1^) spectra was recorded. As per the α-chitin standard, the well-characterized chitin from the spider *Caribena versicolor* was used [43]. The degree of acetylation (DA) was calculated as follows—Equation (1) [44]; and the degree of deacetylation (DD) using Equation (2) [45]:

DA% = [(A_1654_/A_3432_)·100%)]/1.33 (1)

DD% = 100% − DA%(2)

#### 2.3.3. Thermogravimetric Analysis (TGA/DTG)

Qualitative characterization of the isolated chitinous scaffold was performed by thermogravimetric analysis (TGA) using a Jupiter STA 449 F3 analyzer (Netzsch, Selb, Germany). Specimens (~10 mg) were inserted on a thermobalance and heated from 30 to 1000 °C at a heating rate of 10 °C/min in a nitrogen atmosphere. Additionally, a DTG curve was plotted to better visualize the process of thermal decomposition.

#### 2.3.4. Dynamic Mechanical Analysis

The mechanical (compressive) properties of the isolated scaffolds were investigated by means of monotonic compression tests. Specimens were prepared as cylinders with diameter ca. 9 mm and height ca. 1 mm. Monotonic compression tests were performed by dynamic mechanical analysis (DMA) using a Q800 instrument (TA Instruments, New Castle, DE, USA). Over the tests, specimens were completely immersed in demineralized water [46]. Samples were preloaded to 0.001 N and compressed at a constant strain rate of 5%/minute. The compressive modulus was calculated as the ratio between compressive stress and strain in the linear portion of the curve (at 20% of total strain), and the area below the stress–strain curve represented specific energy absorbed during deflection of the sponges.

#### 2.3.5. Microcomputer Tomography (μCT)

The μCT image acquisition of the sponge fragment (around 5 × 5 × 4 mm) was performed in wet conditions. Prior to scanning, samples were partially dehydrated and then dipped into sunflower oil to improve x-ray image contrast. Scanning was performed using a microfocussed X-ray tomographic system (MICRO XCT-400, Xradia–Zeiss, Pleasanton, CA, USA) with the following parameters: 40 kV voltage, 10 W power, no filter material, 0.16° rotation step in an angle interval of 184°. The voxel size was 5 × 5 × 5 μm^3^. The data processing, image analysis and 3D reconstruction of the scanned samples were performed with Avizo Fire (Thermo Fischer Scientific, Hillsboro, OR, USA).

Porosity was calculated by simple voxel counting in the 3D segmented image. Local pore diameter and fiber thickness were calculated using a model independent sphere filling technique [47]. Outcomes are expressed as mean ± standard deviation (SD).

#### 2.3.6. Scanning Electron Microscopy (SEM)

For SEM examination, the scaffolds with imposed cells of the studied lines were fixed in 2.5% glutaraldehyde (Serva Electrophoresis, Heidelberg, Germany) diluted in cacodylate buffer (0.2 M, pH 7.4, Serva Electrophoresis). After 1 h, the samples were washed three times in cacodylate buffer for 5 min at room temperature and postfixed for 1 h at 4 °C in 1% osmium tetroxide (Serva Electrophoresis) diluted in cacodylate buffer. The samples were next bathed three times with cacodylate buffer for 5 min. Dehydration of the samples was performed by increasing concentrations of ethanol (Stanlab, Lublin, Poland) for 15 min in each solution (50%, 70%, 80%, 96%) at 4 °C. Subsequently, the studied cell lines were incubated in absolute alcohol three times for 15 min at room temperature. Finally, the specimens were transferred to pure acetone, air-dried, and covered with 30 nm of gold in a high-vacuum sputter coater (Edwards, Burgess Hill, United Kingdom). Observations were taken with a JSM-6610A scanning electron microscope (JEOL, Tokyo, Japan) using 20 kV accelerating voltage and a secondary electron detector, revealing topography contrast.

#### 2.3.7. Transmission Electron Microscopy (TEM)

To examine the cell morphology seeded on pure chitin scaffolds, the ultrastructure of the cell lines was checked using the TEM method. Chitin scaffolds with the imposed cells were placed in the cultured media. Next, the medium was discarded, and a 3.6% glutaraldehyde solution freshly prepared in 0.2 M cacodylate buffer was added (25 min, room temperature). In the next step, the fixative was rinsed with 0.1 M cacodylate buffer (4 × 15 min) and the samples were left in the same buffer overnight at 4 °C. The cells were then subjected to secondary fixation in 1% osmium tetroxide diluted in 0.1 M cacodylate buffer (1 h, room temperature), followed by washing of the samples in 0.1 M cacodylate buffer (3 × 5 min). Next, the material was passed through a series of graded ethyl alcohol solutions (30%, 50%, 70%, 10 min each, room temperature) and left overnight at 4 °C in a 70% solution. The next day, dehydration was continued in solutions of 80% and 90% ethanol, then in a mixture of 90% ethanol/90% acetone (1:1). Afterward, the cells were passed through a graded series of acetone (90%, 95%, and 100%). After dehydration, the material was incubated with epoxy resin mixed with pure acetone in a ratio of 1:3 (20 min), then in mixtures of 1:1 (60 min) and 3:1 (60 min), all at room temperature, and finally refrigerated overnight in pure epoxy resin. Finally, the resin-saturated material was transferred to specimen boxes (flat embedding molds; Pelco, Ted Pella, Redding, CA, USA) and flooded with epoxy resin with a catalyst to facilitate its polymerization. The samples were kept in the oven at 60 °C for 7 days. Subsequently, epoxy blocks with the embedded scaffolds were cut on a Power Tome XL ultramicrotome (RMC, Tucson, AZ, USA) with a Histo diamond knife (Diatome, Nidau, Switzerland) into semithin sections of 600 nm thickness. After drying on a heating plate, the sections were stained with a dye solution: toluidine blue (Serva Electrophoresis) and anhydrous sodium carbonate Na_2_CO_3_ (Alchem, Toruń, Poland), and closed with a Euparal mounting agent (Roth, Mannheim, Germany). The obtained microscope slides allow careful selection of the studied cell lines for making ultrathin sections (thickness ranging between 60 and 70 nm) with the use of an Ultra 45° diamond knife (Diatome) for TEM documentation. The ultrathin sections were transferred to rhodium–copper grids (Maxta form, 200 mesh, Ted Pella, Redding, CA, USA), contrasted with uranyl acetate and lead citrate trihydrate (Serva Electrophoresis), followed by washing of the grids three times in demineralized water. The grids were stored on a petri dish for 1 h to dry before being viewed under a JEM-1011 transmission electron microscope (JEOL, Tokyo, Japan) operating at 80 kV. Electronograms were collected with the use of the TEM imaging platform iTEM1233 equipped with a Morada Camera (Olympus, Münster, Germany) at magnifications ranging from 3 to 20 K.

### 2.4. Biological Evaluation

#### 2.4.1. Cell Lines

The mouse normal fibroblasts cell line Balb/3T3 (American Type Culture Collection ATCC^®^, Old Town Manassas, VA, USA), the normal human dermal fibroblasts line (NHDF) (Lonza, Basel, Switzerland), the human epidermal keratinocyte line (HaCaT) (DKFZ, Heidelberg, Germany) [48] and the human neuroblastoma cell line (SH-SY5Y) (ATCC^®^) were used in the study. The Balb/3T3 and HaCaT lines were cultured with DMEM medium (Lonza) with 10% foetal bovine serum (FBS) and 1% L-glutamine with penicillin and streptomycin solution (Sigma-Aldrich^®^, St. Louis, MO, USA). NHDF was grown in FGM^TM^ Fibroblast Growth Medium BulletKit^TM^ (Lonza). SH-SY5Y was cultured with F-12 medium (Lonza) supplemented with 10% FBS (Sigma-Aldrich^®^) and 1% L-glutamine with penicillin and streptomycin solution (Sigma-Aldrich^®^). Cell culture was maintained in 5% CO_2_ at 37 °C and 95% humidity. The cells were assessed twice a week, and the fresh medium was changed or passaged if confluence was approximately 70%.

#### 2.4.2. Determination of Cytotoxicity

The scaffolds were evaluated in accordance with the direct method of cytotoxicity evaluation described in ISO 10993-5: 2009 “Biological evaluation of medical devices—Part 5: In vitro cytotoxicity studies” [49,50]. The Balb/3T3 cells were cultured in a 6-well plate (1.0 × 10^5^ cells per well). After 24 h of culture, a scaffold was immersed in the culture medium and the experiment was carried out for 24 h. Using an Olympus BX51 fluorescence microscope inverted contrast-phase light microscope (Olympus, Tokyo, Japan), morphological changes of cells surrounding the scaffold in the culture were observed. The evaluation was also carried out in a control culture, without contact with the evaluated material.

#### 2.4.3. Determination of Cell Adhesion

Mouse fibroblasts (Balb/3T3), normal human dermal fibroblasts (NHDF), and human epidermal keratinocyte (HaCaT) cell cultures were carried out on isolated scaffolds with dimensions of approx. 0.5 × 0.5 × 0.4 cm, with cell numbers of 1.0 × 10^5^ (24-h cell culture) and 5.0 × 10^4^ (7-day cell culture) in 3 mL of complete medium. Cultures were conducted in the complete culture medium described above, in 6-well plates under a controlled gentle rocking platform which allowed verification of the real adhesion of cells on the scaffold surface. Next, the cells were visualized using the following methods.

*Crystal Violet staining*: Cells were fixed: washed twice with cold PBS (Lonza) and then incubated for 10 min in cold methanol (−20 °C) (Chempur^®^, Piekary Slaskie, Poland). After that, cells were incubated for 10 min in a solution of 0.5% Crystal Violet in 25% methanol (Sigma-Aldrich^®^) [51]. The Crystal Violet was then washed several times in water, and the cell adhesion to the scaffold surfaces was assessed using an Olympus CKX53 inverted contrast-phase microscope.

*Neutral Red staining*: The medium with cells cultured with scaffolds was removed and a fresh culture medium with 40 μg/mL of Neutral Red (Sigma Aldrich^®^) was added [52]. After 3 h of incubation under standard conditions (37 °C, 5% CO_2_) the cell adhesion to the scaffold surfaces was assessed using an Olympus CKX53 inverted contrast-phase microscope and an Olympus BX51 fluorescence microscope.

#### 2.4.4. Experimental Model for Neuronal Cells

Two types of 12-well plates were used in experiments—plates provided from the producer, and plates with the modified surface of the culture wells with collagen. The modification was carried out using collagen type I solution at a concentration of 0.01% (*w*/*v*) overnight at 4–8 °C. Before use, the plates were exposed to UV light for 30 min for sterilization. The scaffold was cut into 0.5 × 0.5 × 0.4 cm fragments and then used in experiments in two ways. SH-SY5Y cells were seeded at a density of 50,000 cells/well directly onto the material (chit.1), or the scaffold without cells was immersed into a well with the previously seeded cells (chit.2). For the cells’ adherence to the scaffold, 50,000 cells were seeded directly onto it in a volume of 100 μL medium. After 30 min of incubation, the solution in the well was diluted to a final volume of 1 mL. Two types of control were used in the experiments. The first control cells were cultured only in complete medium F12 (Compl.) and in the second, in a medium containing retinal acid/NGF (neurite growth factor). Observations and evaluations were carried out at four time points: one day after seeding, and after 3, 5, and 9 days of incubation with retinal acid/NGF. The number of cells in the collected medium that adhered to the scaffold and to wells’ surface were counted. The SH-SY5Y cells were counted on the three different levels of the scaffold. The study involved four independent experiments under repetitive conditions, each of which included three replicates.

##### Detection of Apoptosis

Cell apoptosis was checked using a staining kit provided by Sigma-Aldrich (St. Louis, MO, USA) containing Annexin V conjugated with Fluorescein and propidium iodide. The SH-SY5Y cells were transferred to test tubes and incubated with Annexin V and PI solution in the dark at RT for 20 min. The viability of the culture was assessed by the image-based cytometer Arthur (NanoEnTek, Seoul, Korea) (living, apoptotic and necrotic cells).

##### Analysis of the Number of Cells on the Scaffold

The scaffold was cut into 0.5 × 0.5 × 0.4 cm fragments and then used in experiments in two ways. SH-SY5Y cells were seeded at a density of 5 × 10^4^ cells/well directly onto the material. For the cells’ adherence to the scaffold, 5 × 10^4^ cells were seeded directly onto it in a volume of 100 μL medium. After 30 min of incubation, the solution in a well was diluted to a final volume of 1 mL. Two types of control were used here. The first control cells were cultured only in a complete medium, and the second in a medium containing retinal acid. Observations and evaluations were carried out at four time points: one day after seeding, and after 3, 5, and 9 days of incubation with retinal acid. The number of cells adhering to the scaffold and adhering to the wells’ surface was counted. The SH-SY5Y cells were counted on three different levels of the scaffold. The study involved four independent experiments under repetitive conditions, each of which included three replicates.

##### Statistical Analysis

The obtained results have been analyzed according to generally accepted principles. Initially, the normal distribution was checked using the Shapiro–Wilk test, and equality of variance using Levene’s test. The Kruskal–Wallis test and post hoc analysis have been done.

## 3. Results and Discussion

**Figure 2:**
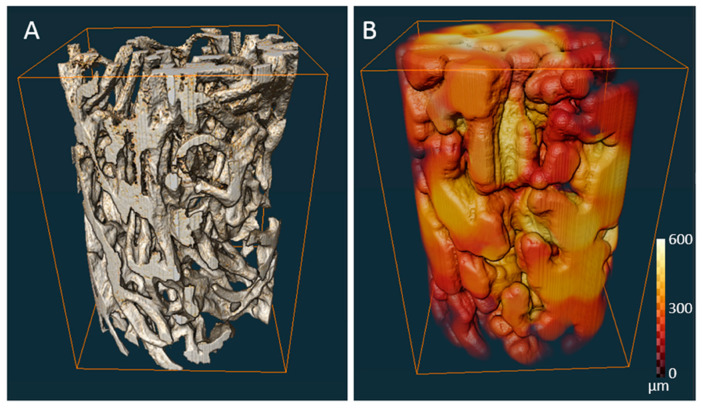
Three-dimensional visualization obtained from μCT analysis of (**A**) chitinous scaffold structure in wet conditions and (**B**) colored maps of pore sizes distribution.

The microstructure of chitin-based scaffold from *A. fistularis* marine sponge skeleton is represented in Figure 1C,D. As excepted, the typical macro-porous morphology of the sponge structure is visible [53]. The polydisperse nature of the interconnected pore sizes is illustrated in Figure 2, where variations of the colors represent pores of different dimensions. The relationship between colors and interconnected pores size is shown by color calibration bars. Moreover, for more details, the main porosity parameters are given in Table 1.

**Figure 3:**
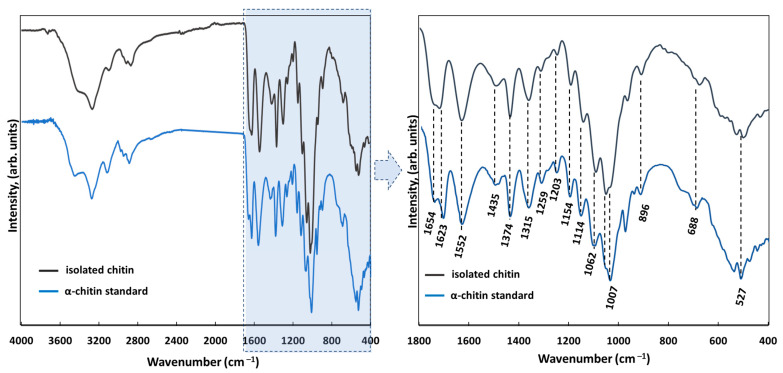
FTIR spectrum of chitin-based scaffold (grey line) obtained from *A. fistularis* sponge as well as an α-chitin standard (blue line) isolated from the *Caribena versicolor* spider’s molt cuticle, fully characterized previously [43].

Figure 3 shows the FTIR measurements of the pure chitinous skeleton from *A. fistularis* (grey line) used as a scaffold for cell culture. Based on our calculations (Equations (1) and (2)), the degree of acetylation (DA) was determined as 79%, which is characteristic of chitin isolated by alkali treatment [44]. The degree of deacetylation (DD) was calculated as 21%, which confirms a highly N-acetylated biopolymer [54]. The spectrum includes a split peak at 1654 cm^−1^, 1623 cm^−1^ corresponding to stretching vibrations of C=O bonds (amide I) in chitin [55]. This characteristic band is associated with the α-chitin polymorph, and more precisely with stretching vibrations from inter- (C=O· · ·H–N) and intramolecular (C=O· · ·HOCH_2_) hydrogen bonding [53]. The existence of α-chitin in the skeleton of the *A. fistularis* marine sponge was proved previously [53]. Moreover, the presence of such signals is characteristic of amide II (νN–H and νC–N) at 1552 cm^−1^, and amide III (CH_2_ wagging) at 1315 cm^−1^. This also established the pure α-chitin existence [7,44,56]. Other bands associated with chitin were observed at 1435 cm^−1^ (CH_2_ bending and CH_3_ deformation), 1374 cm^−1^ (CH bending and CH_3_ symmetric deformation), 1154 cm^−1^ (asymmetric carbon-oxide bridge) [57], 1062 cm^−1^ (C–O–C stretching), 896 cm^−1^ (corresponding to glycosidic linkage and CH stretching vibrations of saccharide rings), and 688 cm^−1^ (OH out-of-plane bending) [44,56,57]. Compared with the α-chitin standard our scaffold did not exhibit any additional signals, which confirms the obtaining of a pure chitinous scaffold.

**Figure 4:**
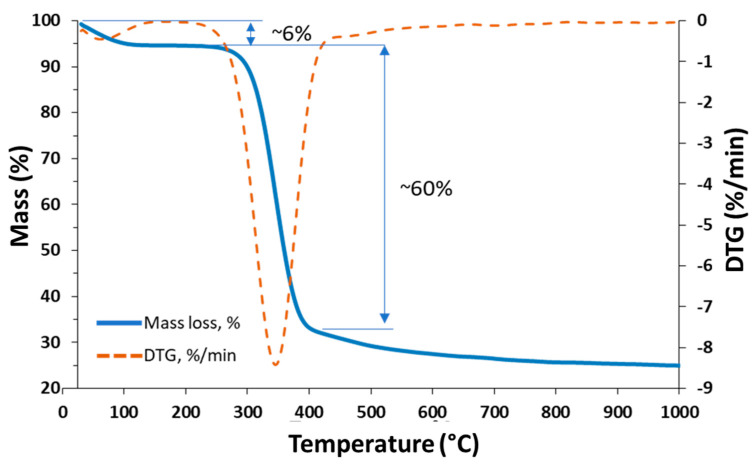
TGA/DTG curve of pure chitinous scaffold isolated from *A. fistularis* marine sponge.

The TGA graphs shown in Figure 4 present two significant regions. The first lies in a temperature range of 80–110 °C, caused by the loss of water molecules physically and chemically bound to the material [58]. The quantity of water lost is estimated at around 6%. The second important mass loss lies in the temperature range 200–400 °C and correspond to the thermal and oxidative decomposition of the α-chitin [56]. The highest rate of decomposition, represented by the maximum peak of the DTG curve, about 8%/min for pure chitin, occurs at a temperature of about 330 °C. The characteristic TGA curve may indicate the presence of a pure chitinous scaffold.

**Figure 5:**
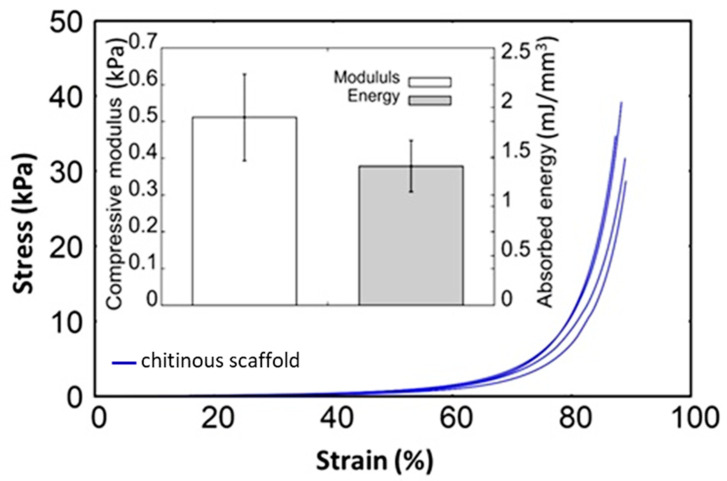
Compression stress–strain response for pure chitin-based scaffolds obtained by alkali-acid based treatment of *A. fistualris* skeleton.

The macro-scale mechanical properties of the α-chitin scaffolds isolated from *A. fistularis* marine sponge were identified here. The Young’s modulus of native soft tissues and organs has been found to range from 0.1 kPa to 1 MPa, depending on the function and tissue type [59,60]. For example, in work present by Přádný et al. [22], authors mentioned, that collagenous layers from biological tissues reflect Young’s modulus range, <1 kPa (nerve tissue), approximately 10 kPa (muscle tissue), and about 100 kPa (hard tissue). Thus, the mechanical properties of scaffolds are among the most important parameters limiting cellular behavior [59]. As reported by Breuls et al., stiffness values for PA hydrogel should possesses, for example, ~0.5 kPa (brain tissue), ~10 kPa (muscle tissue), and >30 kPa (pre-mineralized bone) [61]. Previously, polyacrylamide (PA) hydrogel acting as a cell culture support (~1 kPa) has been successfully used for human fibroblast cultivation. However, Intini et al. used a chitosan-containing 3D-printed scaffold with Young’s modulus ~105 kPa for an in vitro experiment with human skin cells [62]. Figure 5 illustrates the stress–strain curves obtained for scaffolds in a monotonic compressive test. The curves exhibit a non-linear trend without exact yielding points. The low stiffness in the first stage of test growth is very intensive in the second stage, similarly to natural extracellular matrices (ECMs) [63]. The overall apparent compressive modulus was estimated here as ~0.5 kPa. However, the stress is imposed on few fibers within the sample cross-sectional area and could be disturbed by water flow out of the structure. Modulus is a product of proportionality between acting stress and strain, while stress is expressed as force per area. In this case, the apparent modulus of the structure is much lower than the modulus of the fiber bulk. Thus, in future research, the modulus of the fiber bulk should be determined for better understanding.

**Figure 6:**
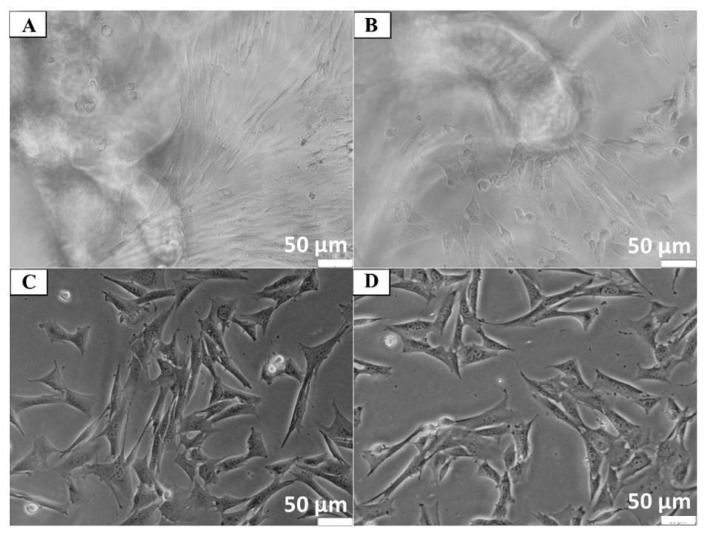
Evaluation of cytotoxicity of chitin scaffolds after 24 h contact with Balb/3T3 cell culture. (**A**) Cells under the scaffold; (**B**) near the scaffold; (**C**) at a distance from the scaffold; (**D**) control culture.

Cytotoxicity studies of chitin scaffolds did not show any changes in Balb/3T3 cell morphology after 24 h incubation under and near the scaffolds, which indicates the material’s lack of cytotoxicity. The morphology of cells incubated with chitin scaffolds was indistinguishable from that of the control cell culture without contact with this material (see Figure 6).

**Figure 7:**
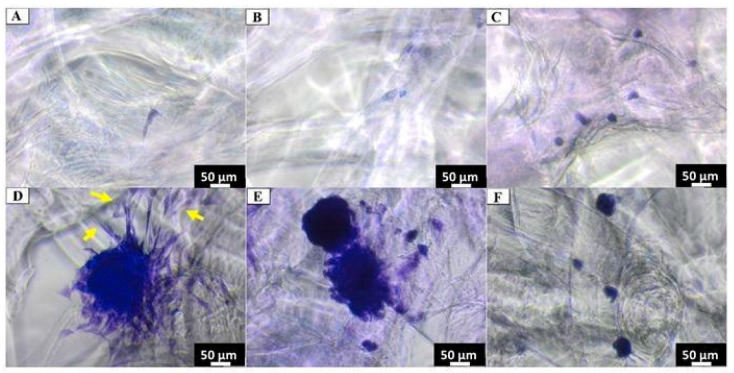
Cell cultures on chitin scaffold surface visualized with Crystal Violet staining (**A**–**C**) after incubation for 24 h and (**D**–**F**) for 7 days. (**A**,**D**)—Balb/3T3; (**B**,**E**)—NHDF; (**C**,**F**)—HaCaT.

Crystal Violet staining showed single cells of Balb/3T3, NHDF and HaCaT adhering to the chitin scaffold surface after 24 h of the experiment. However, after 7 days of incubation, clusters of cells were observed on the chitin scaffold surface, which indicates cell adhesion on the investigated material (Figure 7). Interestingly, cell spreading was observed only after 7 days of incubation (see yellow arrows in Figure 7D). Nevertheless, the round morphology adopted by cells and limited spreading may be associated with the very soft mechanical properties of the obtained constructs and low protein adsorption [61]. In this context, the natural forces generated by the adhering cells probably exceeded the physical interactions between the adsorbed proteins and the chitin matrix. Thus, cells proliferation has been determined in respect to neuronal cells below.

To investigate the biocompatibility of the of the 3D chitin scaffold on the different types of cells involved in wound healing (keratinocytes and fibroblasts), neutral red live-cell staining was performed. Our results show the single cells of Balb/3T3, NHDF, and HaCaT adhering to the chitin scaffold surface after 24 h of incubation. In addition, after 7 days of the experiment, clusters of cells were visible on the chitin scaffold surface under inverse contrast-phase and fluorescence microscopes (see Supplementary Materials Figures S1 and S2).

**Figure 8:**
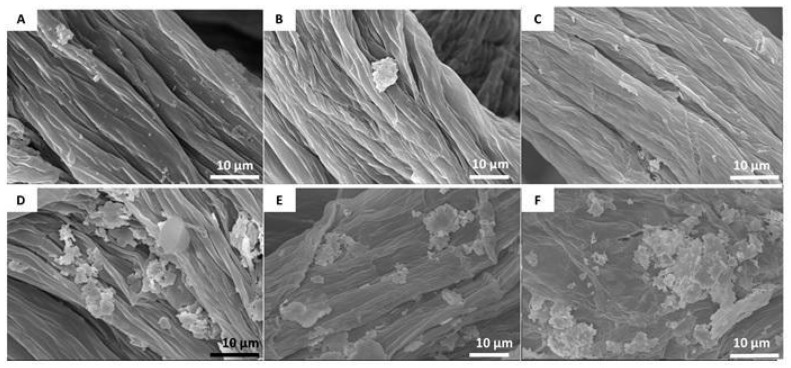
SEM photographs showing cell adhesion on a chitin scaffold surface. Cells after 24 h and 7 days of culture have been shown. (**A**,**D**)—Balb/3T3; (**B**,**E**)—NHDF; (**C**,**F**)—HaCaT. A rise in the number of cells is visible after 7 days compared to the 24 h cell cultures.

To further characterize the Balb/3T3, NHDF, and HaCaT of cells attached to the chitin scaffold, scanning electron microscope images were obtained. Visualization of cell behavior on the chitin scaffolds after 24 h of incubation on the material surface confirmed the previous results. Analysis of cells cultured for 7 days showed clusters of cells on the chitin scaffold surface, which suggests that the cells adhere and proliferate on that surface (Figure 8). Similar cell behavior has been observed previously on a poly(e-caprolactone)/chitosan scaffold [64]. For a comparison with a chitinous scaffold surface before cell seeding, see Figure S3.

**Figure 9:**
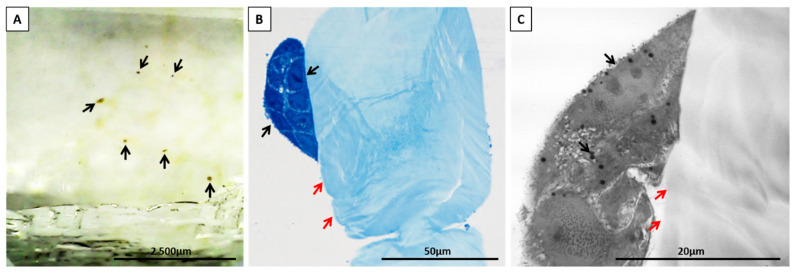
Clusters of HaCaT cells (black arrows) after osmium tetroxide post-fixation (**A**), toluidine blue staining (**B**), and counterstaining with the heavy metal salts (**C**) are attached to the chitin scaffolds (red arrows). A—epoxy resin block; B—semithin section; C—ultrathin section.

**Figure 10:**
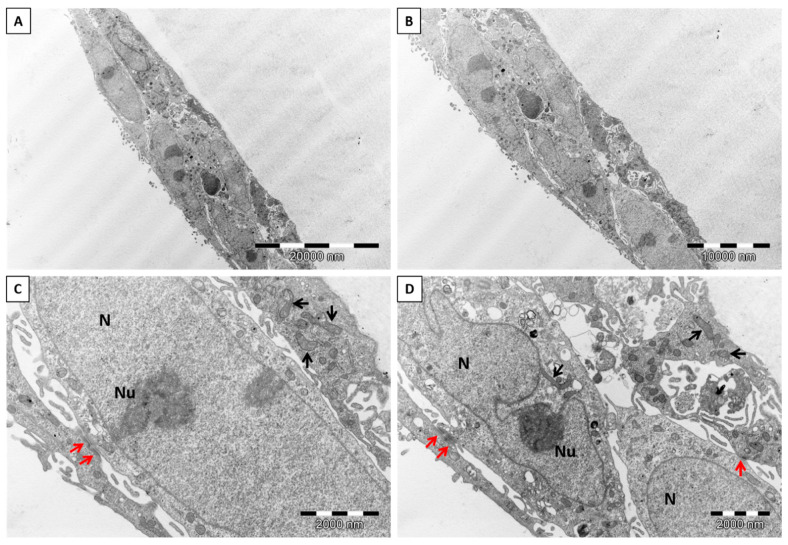
Representative electron microphotographs of HaCaT cell morphology. Nuclei without signs of death are conspicuous, and the well-preserved ultrastructure of organelles is visible. N—nucleus, Nu—nucleolus, black arrows—mitochondria, red arrows—desmosomes. Magnification: (**A**)—3000×; (**B**)—4000×; (**C**,**D**)—20,000×.

The ultrastructure of keratinocytes and fibroblasts was evaluated after enhancing the contrast (counterstaining) of the cell membranes with uranyl acetate and lead citrate, respectively. Both single cells and clusters of cells were visible directly on the surface of the chitin scaffolds or in their vicinity (Figure 9A–C). Keratinocytes were oval or spindle-shaped, with an elongated euchromatic nucleus. The nuclei contained one to three distinct and compact nucleoli and had an irregular nuclear envelope. The cytoplasm was rich in lipid droplets, vacuoles, polyribosomes, scattered sacs of rough endoplasmic reticulum (RER), and intermediate filaments. Elongated or round mitochondria contained lamellar cristae (Figure 10A,B). Furthermore, keratinocytes formed many layers of cells interconnected with each other by well-developed desmosomes (Figure 10C,D). The ultrastructure of the fibroblasts was as follows, the cells contained conspicuous and well-developed Golgi apparatus, abundant vesicles, and multivesicular bodies. Furthermore, the cell membrane of the fibroblasts formed short and stubby microvilli. In comparison to keratinocytes, the cytoplasm contained many vacuoles and more abundant sacs of RER. Round or elongated mitochondria featured lamellar cristae. The oval euchromatic nuclei had indentations in the nuclear envelope and prominent reticular nucleoli (Figure S4A–D). The ultrastructure of both cells, keratinocytes and fibroblasts, was excellently preserved and did not show features of degeneration, which additionally support our results about nontoxicity and biocompatibility of the chitin scaffolds. There were no signs of any of the well-known types of cell death, like chromatin condensation and fragmentation, shrinkage of the cells, plasma membrane rupture or blebbing, cytoplasm swelling, or organelle degradation [65,66]. Moreover, we did not observe any ultrastructural changes in mitochondrial cristae (remodeling or rupture) or in the outer mitochondrial membrane, as well as mitochondrial edema, which would indicate the participation of these organelles in the induction of apoptosis or necrosis [66]. Changes in mitochondrial ultrastructure faithfully reflect the cell’s response to stimuli inducing cell death, such as mitochondrial fission and condensation of cristae [65,66]. Our results can indicate that the presented biomaterial forms a favorable environment for the cells. It may also signal future applications of chitinous scaffolds because keratinocytes are the major cell type in the skin. Furthermore, as estimated, keratinocytes play key roles in wound repair as structural and immune functional factors [67]. Nevertheless, it was most important that we prove the non-toxicity of scaffold and determine the proliferation of the neuronal cells line.

**Figure 11:**
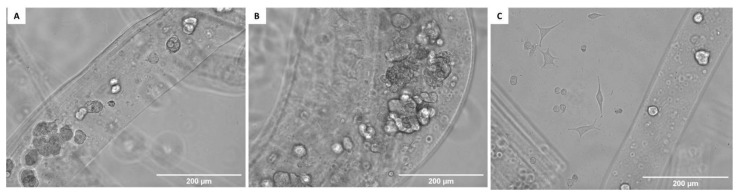
(**A**–**C**) Neuronal cells SH-SY5Y adhering to chitin scaffolds isolated from *A. fistualris* marine sponge skeleton. Scale bar 200 μm.

The Hoechst 33258 staining showed single SH-SY5Y cells adhering to the chitin scaffold surface after 24 h of incubation (Figure 11A). After 5 days of incubation, clusters of cells were observed. These cells grew on top of each other to form a sphere and did not spread over the entire surface of the scaffold, creating a monolayer (Figure 11B). However, after 9 days of incubation, clusters of cells were again observed on the chitin scaffold surface, but these cells also migrated to the surface of the well. After migration of SH-SY5Y cells onto the surface of wells, the cells had normal morphology, and were of polygonal shape with small neurites (Figure 11C). The performed cell apoptosis test showed that the number of apoptotic and necrotic cells did not exceed 7% in total, for all tested cases.

**Figure 12:**
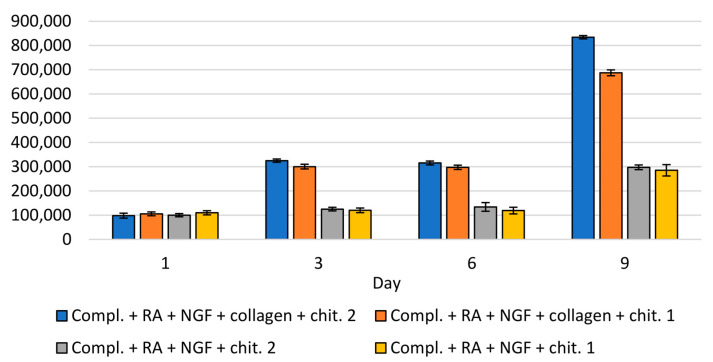
Number of cells SH-SY5Y on chitin scaffold.

The analysis of the number of cells in the collected medium and on the surface of the material was compared to the results obtained for the culture carried out on the modified and unmodified plates. The study was carried out at four time points—in the 1st, 3rd, 5th, and 9th day of breeding. The number of cells in the collected complete medium and medium containing retinal acid is shown in Figure S5, taking into account the culture on modified and unmodified plates. Examination of the influence of the material on modified and unmodified plates was also achieved using the collected medium. In cultures carried out on modified plates, comparing the number of cells in the collected medium on days 3, 6, and 9, statistically significantly higher proliferation was observed in cultures with chitin scaffold, compared to control cultures without it (Figure 12).

The number of cells in the collected medium was also compared in terms of the method of plating cells onto chitin scaffold. On days 3, 6, and 9, a statistically significant difference was observed in the number of cells in the collected medium between cases. SH-SY5Y cells were seeded directly onto the material, and when the material was placed on the surface of the medium with cells. Interestingly, when the chitin scaffold was placed on the surface of the medium with cells on plates modified with collagen, the cell proliferation was greater than on unmodified plates (Figure 12). A similar effect was observed when SH-SY5Y cells were seeded directly onto the chitin scaffold and when the surface of plates was modified with collagen type I. The highest increase in the proliferation between the first day of culturing and the third, sixth, and ninth was observed when wells were modified with collagen, and the chitin scaffold was placed on the surface of medium with cells. This led to the conclusion that the chitin scaffold increased the proliferation of SH-SY5Y cells.

During the evaluation SH-SY5Y, a greater increase in migration of SH-SY5Y cells to the surface of chitin was observed than to the surface of the wells (regardless of whether modified or unmodified). In cultures carried out on a surface modified with collagen and with chitin scaffold immersed into the medium, a much larger number of cells was recorded on the surface of scaffold than on the surface of the well, on 9th day: scaffold—average 1900 cells, surface of well modified with collagen—300–500 cells (data not shown). However, in SH-SY5Y cell cultures grown on the modified surface without scaffold, the number of adherent cells was 800–1000 in the field of view. The above results suggest a higher migration of cells to chitin scaffold than to the surface modified with collagen type I (for more details see Supplementary Materials).

Evaluation of the cell number on the chitin scaffold.

**Figure 13:**
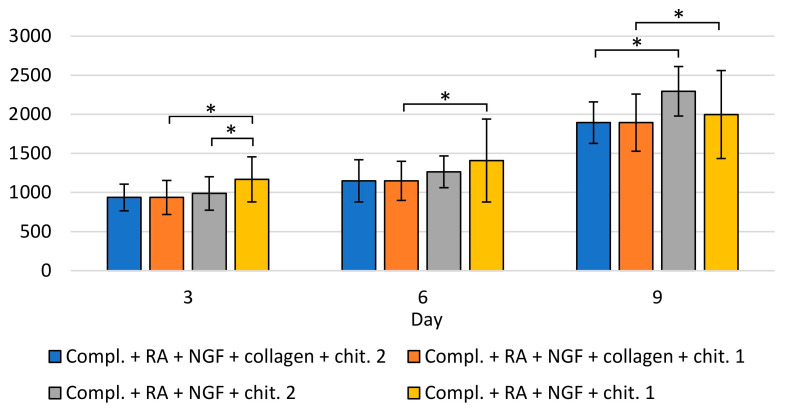
Average number of cells in the field of vision on the microphotographs of chitin scaffold. Statistically significant differences between tested groups: * *p* < 0.05.

A statistically significant increase in the number of cells on the chitin scaffold between all subsequent days of the study was observed regardless of the application method and the surface of the culture plates (modified or unmodified) (Figure 13).

The effect of covering the surface of culture plate wells on the number of cells was examined. When the cells were seeded directly onto scaffold, a significantly higher number of cells on unmodified plates was observed (in days 3, 6 and 9). When material was immersed in the medium with cells, on day 9, it was observed that the number of cells was also higher on unmodified plates. The influence of the material application method was also analyzed. On day 3, there was a significant difference on unmodified plates, depending on the scaffold application method—the number of cells was higher when the cells were seeded directly onto material. On the 6th and 9th day of breeding, no difference was observed, which may indicate that the cells were migrating to the surface of the material. Moreover, there was a noticeably lower number of cells (without statistical significance) on the 9th day on unmodified plates that showed directly on the material (compared to placing the material on the surface of the medium with cells), suggesting that cell differentiation was taking place. There were no statistically significant differences depending on the scaffold application method in the number of SH-SY5Y cells on the chitin scaffold on collagen-modified plates.

Herein, the poriferan chitinous scaffold from *A. fistularis* has been used as a support for the cultivation of major groups of cells taking part in wound healing (keratinocytes and fibroblasts) and neuronal cells. As described above, our study has proven that the chitinous scaffold of marine demosponge origin has no cytotoxic properties, which is in general a desirable feature for materials used in tissue engineering and all biomaterials. The second feature of great importance for a material dedicated to tissue regeneration is its adhesive properties, which enable the cells to cover the surface, and are also crucial for the application of the material in tissue engineering. Our results, obtained with numerous microscopic techniques, show that the murine fibroblast line Balb/3T3, human dermal fibroblas ts (NHDF), human keratinocyte (HaCaT), and mostly human neuronal (SH-SY5Y) cells can adhere and proliferate on the surface of chitin scaffolds. Previously, several time authors proved that chitin-based scaffolds are a promising material for tissue engineering [23,68,69,70,71,72]. In particular, our observations using the TEM technique show that human keratinocytes form clusters on the scaffold, which resemble the cohesive sheets of cells in the stratified epithelium of the skin and could confirm cell adhesion. The cells strongly adhered to the chitin scaffolds and they keep their normal morphology during tissue processing for TEM, although this procedure includes multiply steps. Moreover, inside this cohesive sheet, the keratinocytes were connected to each other by the desmosomes. Desmosomes play a very important function during morphogenesis and differentiation, and a crucial role during cell adhesion [73]. Because no cell swelling or shrinkage was observed, it excluded the loss of adhesion between the cells. These results indicate that poriferan chitin may be potentially useful in skin tissue regeneration. Fibroblasts and keratinocytes are two major groups of cells that take part in wound healing [42,67,74]. This process requires several phases: hemostasis, formation of a fibrin clot, inflammation/granulation, re-epithelialization, and tissue remodeling. In such conditions as venous leg ulcer, diabetic foot, or extensive skin loss, the process of healing can be disrupted and lead to abnormal wound healing [75,76]. The identification of suitable scaffolds on which keratinocytes and fibroblast cells can be seeded to generate functional tissues is an important research goal. Our observations are consistent with the work presented by Noh et al., who seeded normal human keratinocytes and fibroblasts on a chitin-based nanofiber scaffold [69]. An additional preliminary study with neuronal cells (SH-SY5Y) confirmed the adhesion and migration of these cells on the scaffold surface, showing that this material is potentially useful in the field of neuronal regeneration. However, further studies may be carried out to determine the cell adhesion rates in these bioscaffolds, as well as the incubation of cells on the scaffold for an extended time to obtain 3D cell cultures which could be used in structure-based tissue engineering [77]. Nevertheless, functionalization of chitinous scaffold surface is required for improving protein and cell attachment.

## 4. Conclusions

Three-dimensional (3D) cell cultures are essential for bulk cell manufacturing and structure-based tissue engineering. However, their arrangement and preparation are still a challenge due to the lack of a highly biocompatible culture environment for several cell types. Herein, for the first time, chitin-based scaffold obtained from the skeleton of the Verongida sponge *Aplysina fistularis* has been used as a support for the cultivation of murine fibroblasts (Balb/3T3), human dermal fibroblasts (NHDF), human keratinocyte (HaCaT), and human neuronal (SH-SY5Y) cells. Characterization techniques, such as ATR FTIR, TGA, and μCT, clearly indicate that an interconnected macro-porous, thermostable, pure α-chitin scaffold is obtained after alkali–acid treatment of air-dried marine sponge. DMA mechanical testing shows that the chitinous scaffold of poriferan origin exhibits soft mechanical properties like those of a natural extracellular matrix. Nevertheless, future functionalization of scaffold surface is required to improve cell attachment and spreading. The non-cytotoxicity of such a construct is confirmed by a lack of morphological changes in Balb/3T3 cells after 24 h of incubation. Moreover, various staining techniques (Neutral Red, Crystal Violet) and microscopic observations confirmed cell adhesion and proliferation in a 7-day culture. The TEM technique was used for determining the ultrastructure of fibroblasts and keratinocytes cultivated on a chitinous scaffold of marine sponge origin. As observed, keratinocytes form clusters on the scaffold which resemble a skin structure, including the occurrence of desmosomes linking these cells in the cohesive sheet. Our preliminary results show the good proliferation of neuronal cells on the soft chitinous scaffold. This article also describes that cultures of neuronal cells are more likely to migrate to the 3D chitin scaffold than to the surface of type I collagen. Thus, in this study, we reported that three-dimensional chitinous scaffolds are beneficial as they provide a nontoxic, biomimetic environment for various cell types. The use of their naturally formed structure is cost-effective because no additional manufacturing techniques are required for the preparation of centimeter-sized scaffolds.

## Figures and Tables

**Table 1:** Quantitative data on porosity, pore size, and scaffold fiber thickness distributions and average values, obtained by analysis of μCT data.

Porosity (%)	Average Pore Diameter (µm)	Average Fiber Thickness (µm)
79	298 (±43)	98 (±35)

## Data Availability

Data is contained within the article and supplementary material.

## Supplementary Materials

The following are available online at https://www.mdpi.com/article/10.3390/ma14112992/s1. Figure S1. Adhered cells on chitin scaffolds surface visible in fluorescence with Neutral Red staining after incubation (A–C) for 24 h and (D–F) for 7 days. (A,D)—Balb/3T3; (B,E)—NHDF; (C,F)—HaCaT. Figure S2. Adhered cells on chitin scaffolds surface visualized with Neutral Red staining (A–C) after incubation for 24 h and (D–F) for 7 days (D–F). (A,D)—Balb/3T3; (B,E)—NHDF; (C,F)—HaCaT. Figure S3. SEM images of pure chitinous scaffold surface. Figure S4. Representative electron microphotographs of NHDF cell morphology. Nuclei without signs of death are conspicuous and the well-preserved ultrastructure of organelles is visible. N—nucleus, Nu—reticular nucleolus, Mb—multivesicular body, RER—rough endoplasmic reticulum, black arrows—mitochondria, red arrows—Golgi apparatus. Magnification: A—12,000×; B—10,000×; C and D—30,000×. Figure S5. Number of cells in the collected medium (cell cultures without chitin material). Statistically significant differences between tested groups: * *p* < 0.05.
