# Supplementary material for: Naturally Formed Chitinous Skeleton Isolated from the Marine Demosponge Aplysina fistularis as a 3D Scaffold for Tissue Engineering
Source: Materials (Basel). 2021 Jun 1;14(11):2992. doi: 10.3390/ma14112992 (PMC8198059; doi:10.3390/ma14112992)
Supplement: Supplementary file 1 [file materials-14-02992-s001.zip › materials-1184961-supplementary.pdf]

materials

Supplementary materials

Naturally Formed Chitinous Skeleton Isolated from the Marine Demosponge *Aplysina fistularis* as a 3D Scaffold for Tissue Engineering

Tomasz Machałowski [1], Agnieszka Rusak [2,*], Benita Wiatrak [3,4], Katarzyna Haczkiewicz-Leśniak [5], Aneta Popiel [2], Jakub Jaroszewicz [6], Andrzej Żak [7], Marzenna Podhorska-Okołów [5] and Teofil Jesionowski [1,*]

[1] Institute of Chemical Technology and Engineering, Faculty of Chemical Technology, Poznan University of Technology, 60-965 Poznan, Poland; tomasz.g.machalowski@doctorate.put.poznan.pl
[2] Department of Histology and Embryology, Faculty of Medicine, Wroclaw Medical University, Chalubinskiego 6a, 50-368 Wroclaw, Poland; popielaneta1@gmail.com
[3] Department of Pharmacology, Faculty of Medicine, Wroclaw Medical University, J. Mikulicza-Radeckiego 2, 50-345 Wroclaw, Poland; benita.wiatrak@umed.wroc.pl
[4] Department of Basic Medical Sciences, Faculty of Pharmacy, Wroclaw Medical University, Borowska 211, 50-556 Wroclaw, Poland
[5] Department of Ultrastructural Research, Faculty of Medicine, Wroclaw Medical University, Chalubin-skiego 6a, 50-368 Wroclaw, Poland; katarzyna.haczkiewicz@umed.wroc.pl (K.H.-L.); marzenna.podhorska-okolow@umed.wroc.pl (M.P.-O.)
[6] Faculty of Materials Science and Engineering, Warsaw University of Technology, Warsaw 02-507, Poland; jakub.jaroszewicz@pw.edu.pl
[7] Electron Microscopy Laboratory, Faculty of Mechanical Engineering, Wroclaw University of Science and Technology, Wybrzeze Wyspianskiego 27, 50-370 Wroclaw, Poland; andrzej.zak@pwr.edu.pl
* Correspondence: agnieszka.rusak@umed.wroc.pl (A.R.); teofil.jesionowski@put.poznan.pl (T.J.)

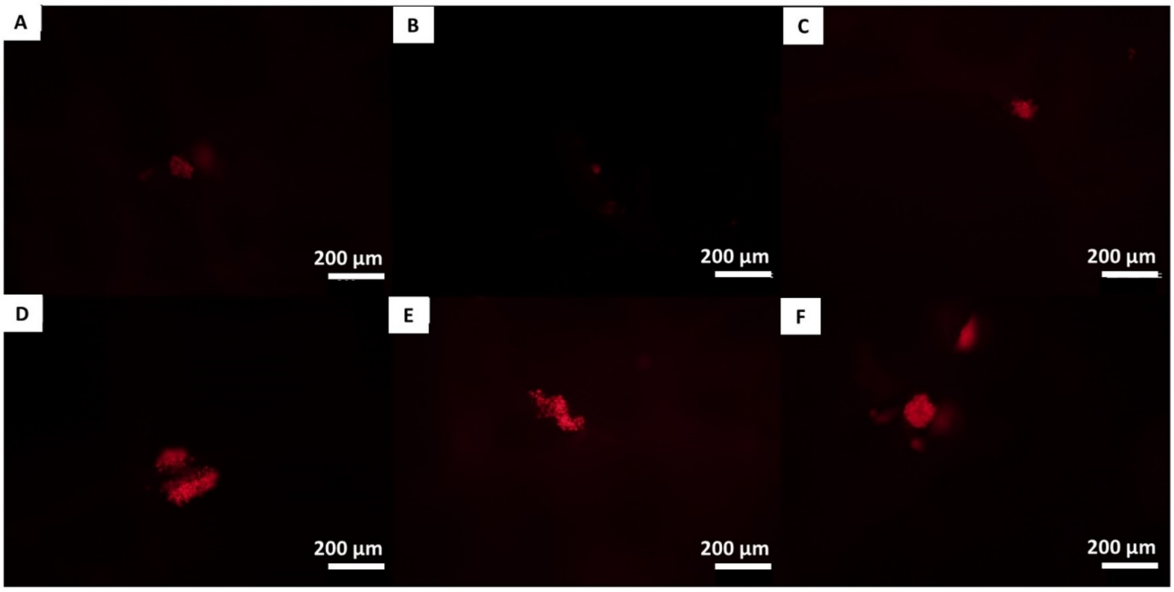

Figure S1. Adhered cells on chitin scaffolds surface visible in fluorescence with Neutral Red staining after incubation (**A**–**C**) for 24 h and (**D**–**F**) for 7 days. (**A**,**D**)–Balb/3T3; (**B**,**E**)–NHDF; (**C**,**F**)–HaCaT.

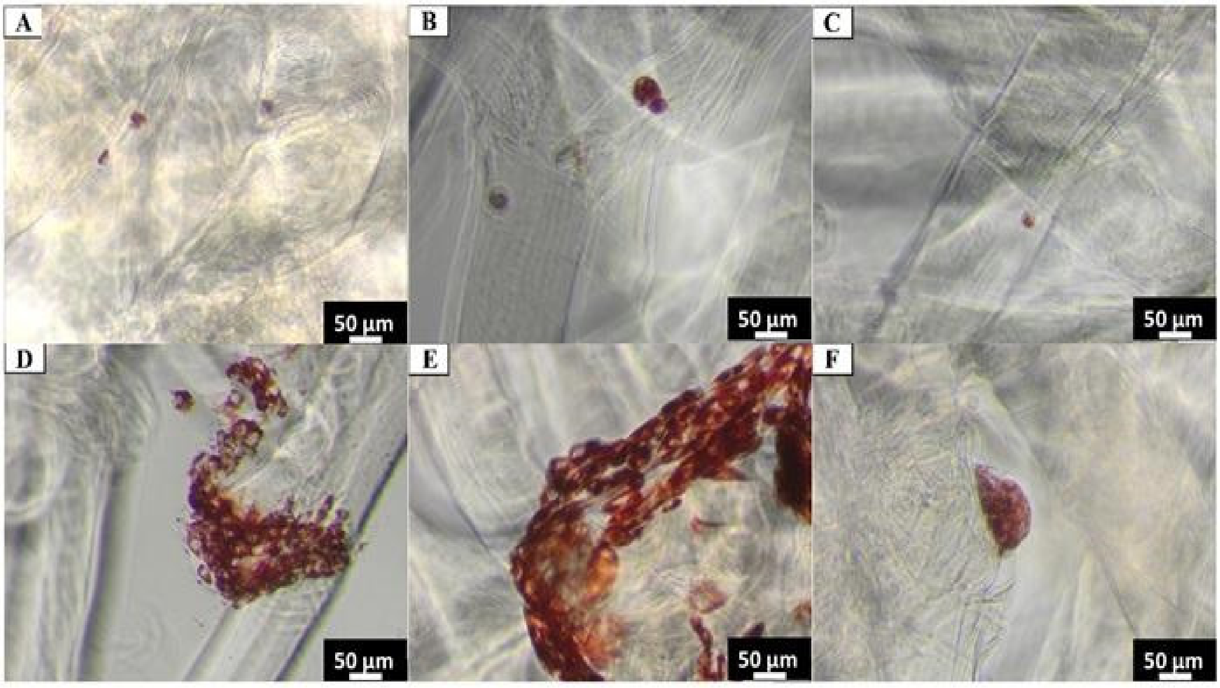

Figure S2. Adhered cells on chitin scaffolds surface visualized with Neutral Red staining (**A**–**C**) after incubation for 24 h and (**D**–**F**) for 7 days. (**A**,**D**)–Balb/3T3; (**B**,**E**)–NHDF; (**C**,**F**)–HaCaT.

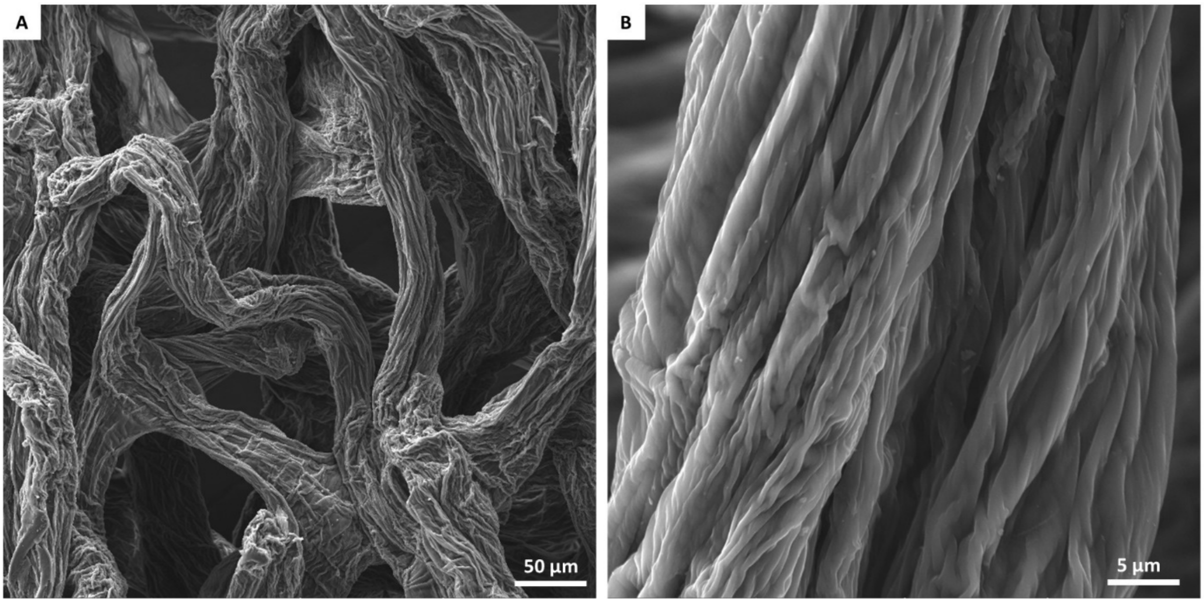

Figure S3. SEM images of pure chitinous scaffold surface.

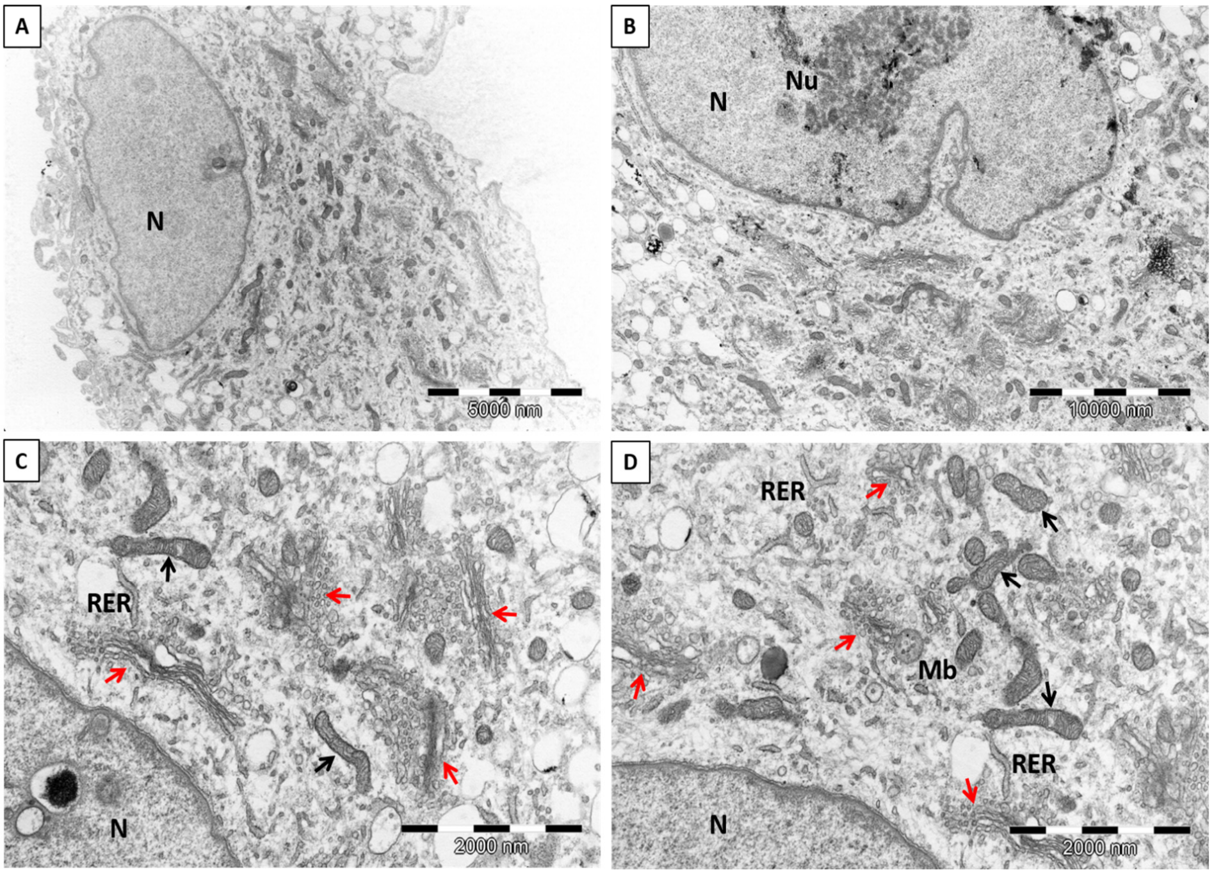

Figure S4. Representative electron microphotographs of NHDF cell morphology. Nuclei without signs of death are conspicuous and the well-preserved ultrastructure of organelles is visible. N—nucleus, Nu—reticular nucleolus, Mb—multivesicular body, RER—rough endoplasmic reticulum, black arrows—mitochondria, red arrows—Golgi apparatus. Magnification: A—12,000×; B—10,000×; C and D—30,000×.

The increase in cell proliferation was greater on unmodified plates. On plates modified with collagen, the cells became adherent to the surface, reducing the intensity of cell divisions. We observed that incubation with retinal acid decreased the proliferation of SH-SY5Y cells over time—especially on the 9th day of incubation with retinal acid occurred a statistically significant reduction in the number of cells in the collected medium regardless of the type of well surface. Cells treated with retinal acid begin to differentiate, and the proliferation phase is stopped. It was observed mainly on the surface previously modified with collagen—the cells adhered to the surface, and after adding the retinal acid, the cells tended to differentiate (Figure S5).

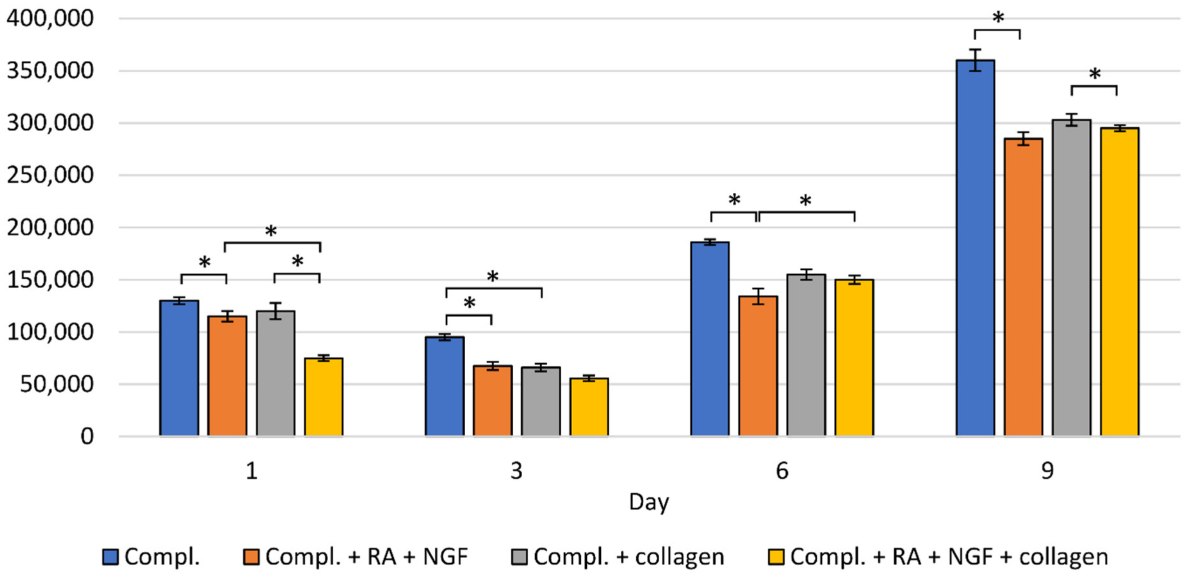

Figure S5. Number of cells in the collected medium (cell cultures without chitin material). Statistically significant differences between tested groups: * $p < 0.05$.

Evaluation of the Effect of Chitin Scaffold on SH-SY5Y Cells Migration

The photos of SH-SY5Y cells stained with Hoechst 33258 on the chitin scaffold and the well surface were analyzed (results not shown). It was observed that SH-SY5Y cells did not adhere to the surface of the unmodified plates (the number of cells in the field of vision–14 69). In contrast, in the case of surface modified with collagen type I, the cells underwent adhesion to collagen and after culturing with the medium containing retinal acid became differentiated. In the microscopic assessment of cells on the chitin scaffold, it was also found that the cells underwent adhesion to the surface of material.
